# Morphological, Histochemical and Immunohistochemical Characterisation of the Penis in Free‐Ranging Giant Anteaters (*Myrmecophaga tridactyla*)

**DOI:** 10.1111/ahe.70057

**Published:** 2025-08-06

**Authors:** Fernanda Barthelson Carvalho de Moura, Victor Gustavo Santos Môra, Natalia Camargo Faraldo, Gabriel Correa de Camargo, Carlos Roberto Teixeira, Maria Valeria de Toledo Rodovalho, Tatiane Terumi Negrão Watanabe, Daniel Felipe Barrantes Murillo, Noeme Sousa Rocha, Carlos Eduardo Fonseca‐Alves

**Affiliations:** ^1^ School of Veterinary Medicine and Animal Science São Paulo State University (UNESP) Botucatu Brazil; ^2^ Institute of Health Sciences Paulista University (UNIP) Bauru Brazil; ^3^ Antech Diagnostics, Mars Petcare Science and Diagnostics Fountain Valley California USA; ^4^ Department of Veterinary Pathobiology, College of Veterinary Medicine Oklahoma State University Stillwater Oklahoma USA

**Keywords:** anteater, background lesion, histopathology, immunohistochemistry, male genitalia

## Abstract

The giant anteater ( *Myrmecophaga tridactyla* ) belongs to the superorder Xenarthra and is distributed throughout Central and South America. This animal is listed as ‘vulnerable’ on the International Union for Conservation of Nature's Red List of Threatened Species. Studies on the reproduction of this species are essential because of its peculiarities; however, there is a lack of information on its reproductive aspects and the biotechnologies that can be applied to it. Morphological and histopathological descriptions of the reproductive organs of *Myrmecophaga tridactyla* are fundamental for determining the general characteristics that could improve our understanding of reproductive disorders in this species. Therefore, this study aimed to perform morphological and histochemical characterisation of the penis of *Myrmecophaga tridactyla* . For this purpose, we collected six postmortem samples of giant anteater penises. The penis of the giant anteater has no foreskin, two erectile bodies, a paired corpora cavernosa, corpus spongiosum and urethra. Type I (red) and type III (yellow‐green) collagen fibres were distributed throughout the stroma and erectile bodies of the penis. Periodic acid‐Schiff (PAS)‐positive staining was observed in the epithelial cells at the base of the hair follicles, and immunohistochemical immunolabelling for androgen receptors (AR) and oestrogen receptors (ER) was detected in all cells of the transitional epithelium of the penile urethra. These findings suggest that penile urethra cells are sensitive to oestrogen and progesterone.

## 1 Introduction

The giant anteater ( *Myrmecophaga tridactyla* ) belongs to the superorder Xenarthra and is distributed in Central and South America, where it faces several threats, such as predatory hunting, forest fires and blunt trauma resulting from wildlife‐vehicle collisions (Miranda et al. 2014; Vidolin 2009; Medri et al. 2003). According to the International Union for the Conservation of Nature Red List of Threatened Species, concern has been expressed for the conservation of giant anteaters, as they are considered ‘vulnerable’ (Miranda et al. 2014).

The lack of government conservation programmes (IUCN Red List of Threatened Species 2014; Venancio et al. 2018) and slow reproductive cycle in the wild (Maia 2002; Medri et al. 2003) influence its conservation. As xenarthrans are known to have peculiarities in their ecology and evolution (Gaudin and Croft 2015), studies on their reproduction are critical. The lack of information on the reproduction of these species and the challenges of reproductive biotechnology hinder breeding (Miranda 2015).

A detailed morphological and histochemical description of the reproductive organs of these species is essential for determining their normal features and providing valuable information for future studies focusing on giant anteater conservation. The penis is a reproductive organ with relevant anatomical variations among animal species, including giant anteaters (Mcinnes 2012; Mcinnes and Scudamore 2014; Samuelson 2007; Fromme et al. 2021; Miranda 2014). Some studies have described the structure of the genital organs of this species (Moura et al. 2021). For example, one study described the anatomical macroscopic appearance of the penis, in which giant anteaters presented with a short and conical penis (Fromme et al. 2021).

Fromme et al. (2021) also described the histology of the giant anteater penis, describing the erectile tissue in two parts (dorsal corpora cavernosa and dorsal corpus spongiosum). However, histochemical and immunohistochemical markers have not yet been identified. The limited macroscopic and microscopic descriptions of the giant anteater male reproductive system have affected the development of new strategies for the reproduction of this species (de Moura et al. 2024).

In histological research, different histochemical and immunohistochemical techniques can be used to improve our knowledge of organ structure. The periodic acid‐Schiff (PAS) reaction is essential in histological research because it selectively stains carbohydrates, glycoproteins and mucosubstances, allowing detailed tissue analysis (Wolner et al. 2023). Conversely, immunohistochemistry facilitates the identification of a specific protein within tissue by eliciting an immune response against a particular antibody. Androgen receptor expression can be assessed using immunohistochemistry, and this receptor regulates the growth, differentiation and function of the penis in different species (Chen and Renfree 2020; Khanna et al. 2022; Cunha et al. 2014). AR expression influences erectile physiology and tissue integrity via testosterone‐mediated signalling (Yang et al. 2022). Alterations in receptor activity may contribute to developmental abnormalities or dysfunction (Liu and Jiang 2023).

In different species, such as the spotted hyena ( *Crocuta crocuta* ) (Cunha et al. 2014) and the marsupial tammar wallaby (Chen and Renfree 2020), androgen receptor (AR) expression has been assessed to understand the role of testosterone in erection in both species. Moreover, young and adult subjects present different expression patterns, since AR is more highly expressed after puberty, and assessing AR expression in young and adult subjects helps understand the dynamics of testosterone expression and reproductive status. Another important marker of male reproduction is oestrogen receptor expression (ER). ER and aromatase are widely distributed across multiple penile structures and cell types, including erectile tissues, urethral epithelium, vascular components and neurons, highlighting the intricate role of oestrogen and its receptors in penile function (Lazari et al. 2009; Mowa et al. 2006). Understanding this complexity is crucial and necessitates leveraging available resources such as animal models and human cases of ER or aromatase deficiency. The use of advanced and highly sensitive techniques is essential for further exploration (Mowa et al. 2006).

Given the importance of understanding the histological and physiological reproductive aspects of giant anteaters, this study aimed to provide microscopic, histochemical and immunohistochemical descriptions of the penis of the giant anteater.

## 2 Material and Methods

### 2.1 Ethics Statement

This study was approved by three Brazilian committees responsible for conducting wildlife research: the System for Genetic Heritage and Associated Traditional Knowledge (#C1018E9), the Chico Mendes Institute for Biodiversity Conservation (#7685‐1), and the São Paulo State University Committee on the Use of Animals in Research (177/2020).

### 2.2 Sample Collection and Morphological Analysis

We examined the penises of six giant anteaters that underwent postmortem examinations at São Paulo State University (UNESP, Brazil) from January 2000 to December 2021 and died due to wildlife‐vehicle collisions. Information on each animal, including body weight, is presented in Table 1. Complete signalling, clinical signs and medical histories were collected and analysed to identify any risks or predisposing factors.

**TABLE 1:** Giant anteater information and background lesions.

Identification	Age	Weight
Subject 1	Young	19 kg
Subject 2	Young	22 kg
Subject 3	Adult	38 kg
Subject 4	Adult	40 kg
Subject 5	Adult	37 kg
Subject 6	Adult	35 kg

The sexual maturity of the free‐living giant anteaters was estimated based on the criteria reported by Fromme et al. (2021) and Tanagho and Lue (2014), resulting in two groups: adult and young animals. Adult animals weighed at least 30 kg and were sexually mature. The young animals ranged in age from 10 months to years.

### 2.3 Histochemistry

For histochemical analysis, representative tissue samples were fixed in 10% buffered formalin and embedded in paraffin for routine histological examination. Afterwards, 4‐μM thick tissue sections were stained with haematoxylin and eosin and PAS for morphological evaluation; 8‐μm tissue sections were stained for picrosirius red to assess structural findings. Penile morphology was analysed according to the criteria established by Fromme et al. (2021) and Rossi et al. (2012). The present study used an Opticam 0700S microscope and an AxionCamER5S to obtain the images for histological assessment.

### 2.4 Immunohistochemistry

Formalin‐fixed paraffin‐embedded (FFPE) 3‐uM thick tissue sections were placed on charged slides (Starfrost; Knittel, Bielefeld, Germany). For antigen retrieval, the slides were incubated in citrate solution (pH 6.0) in a pressure cooker (Pascal; Dako, Carpinteria, CA, USA) for 45 min. Endogenous peroxidase was blocked with hydrogen peroxide 8% diluted in methyl alcohol for 15 min and treated with skim milk 8%, at room temperature. The sections were incubated overnight at 4°C with primary antibodies anti‐AR (1:50; cloneab77557; Abcam, UK) and ER (1:50; clonePPG5/10; Envision, Dako, US). A polymer detection system (EnVision; Dako, Carpinteria, CA, USA) was used as the secondary antibody, and immunoreactive cells were visualised by colorimetric detection (3,30‐diaminobenzidine). The sections were counterstained with Harris haematoxylin (Dinamica‐Diadema, Diadema, Brazil). Canine penis was used as a positive control for all the antibodies. Immunoglobulin species from the primary antibodies were used as negative controls. The present study used an Opticam 0700S microscope and an AxionCamER5S to obtain the images for immunohistochemical assessment.

## 3 Results

### 3.1 Gross Morphology

**FIGURE 1:**
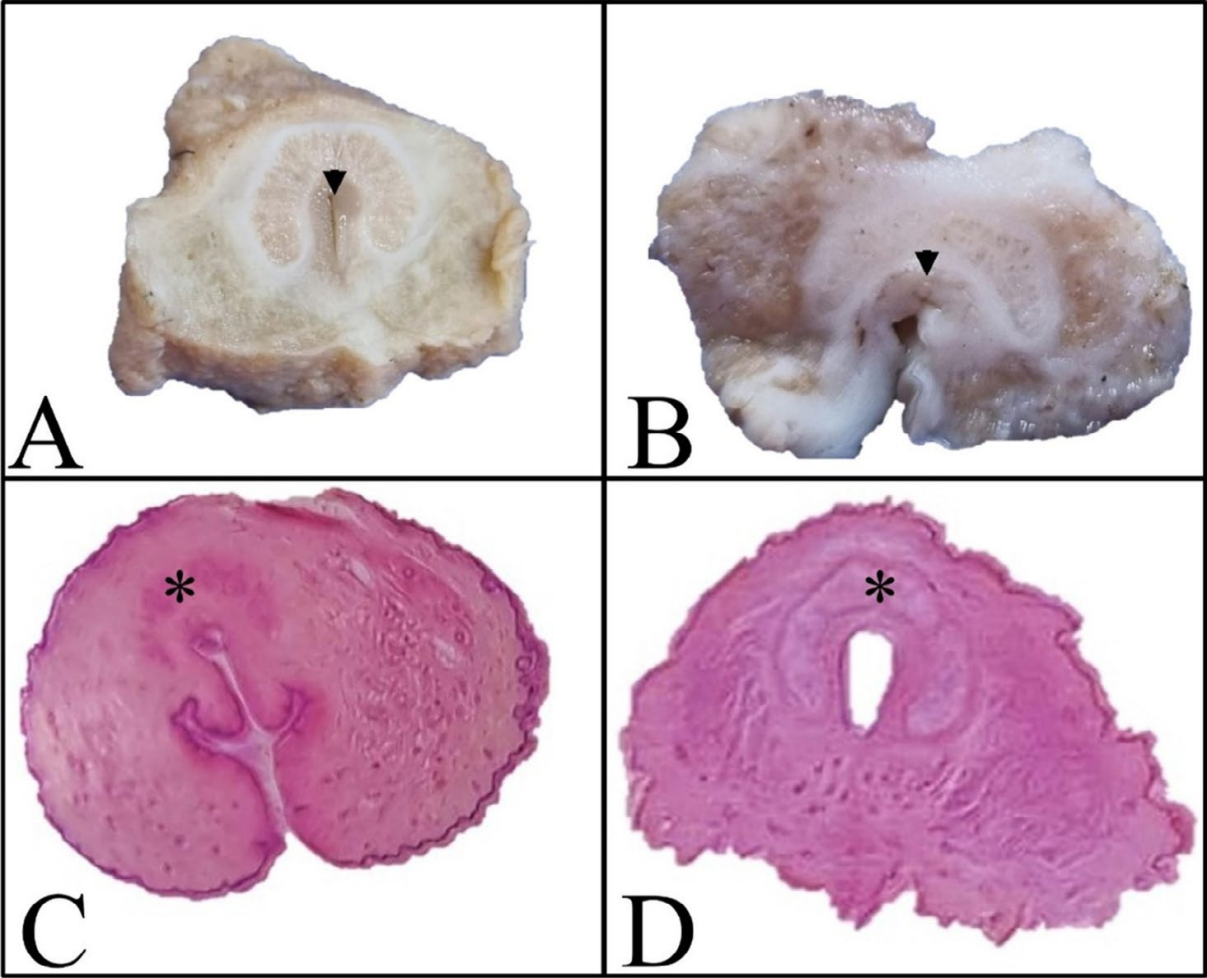
Comparison between gross and digital subgross images of the penis and vulva of adult and juvenile giant anteaters. (A) Cross‐section of the formalin‐fixed penis showing the horseshoe impression surrounding the penile urethra (black arrow). (B) The horseshoe impression was absent in the female vulva. (C, D) Cross section of haematoxylin and eosin (H&E)‐stained penis demonstrating the less developed horseshoe impression (black asterisk) in young (D) than in adult (C) subjects.

After postmortem examination, three penis and one vulva samples from adults (> 2 years old) and two penis samples from young individuals (< 2 years old) were assessed macroscopically and microscopically (Table 1). The gross normal morphology of the penis in adults was conical and horseshoe‐shaped (Figure 1A).

Absence of a horseshoe impression and the presence of developing erectile bodies and corpora cavernosa were noted in the young specimens (Figure 1C,D).

### 3.2 Microscopic Evaluation

**FIGURE 2:**
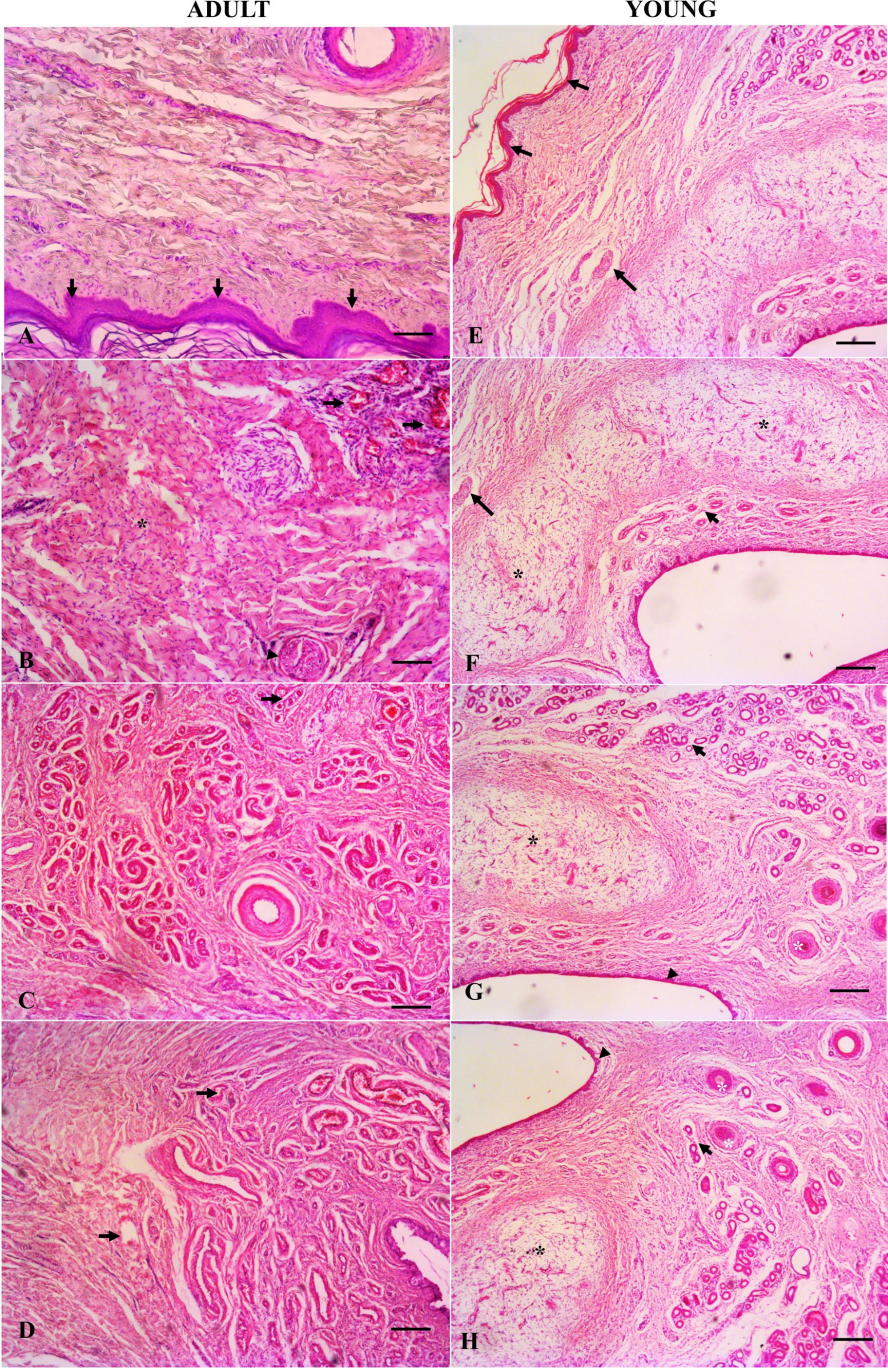
Histological evaluation of giant anteater penis. (A) The penis is lined by keratinised epithelium (black arrow) in adults (H&E, bar = 200 μm). (B–D) Dense connective tissue (black asterisk) with nerves (arrowhead) and blood vessels (black arrow) is distributed around the organ's parenchyma (H&E, bar = 100 μm). (E) The penis is lined by keratinised stratified epithelium (black arrow) in the young specimen (H&E, bar = 200 μm). (F–H) Dense connective tissue with nerves (large black arrow), follicle (white asterisk) and blood vessels (small arrow), and the two erectile bodies (black asterisks) of a young giant anteater surrounding the urethra (bar = 100 μm).

The penis of the giant anteater was covered by a thin layer of epidermis consisting of keratinised stratified squamous epithelium without a foreskin. The tissue adjacent to the epidermis was a dense connective tissue stroma containing blood vessels, nerves and hair follicles distributed around the periphery of the organ. Histologically, the penis of the giant anteater is composed of two erectile bodies. A pair of corpora cavernosa is located ventrally, and the corpus spongiosum surrounds the urethra dorsally. The pair of corpora cavernosa has a horseshoe shape, organised by a layer of dense connective tissue ventrally, blood vessels, trabeculae and smooth muscle fibres distributed throughout the parenchyma. The concavity of the corpora cavernosa involves the corpus spongiosum and urethra dorsally. The corpus spongiosum forms around the penile urethra and is located dorsally to the corpus cavernosum. It is also an erectile tissue composed of loose connective tissue and muscle fibres and contains cavernous spaces lined with endothelium (Figure 2). The urethra is located centrally and is lined by a stratified transitional epithelium.

**FIGURE 3:**
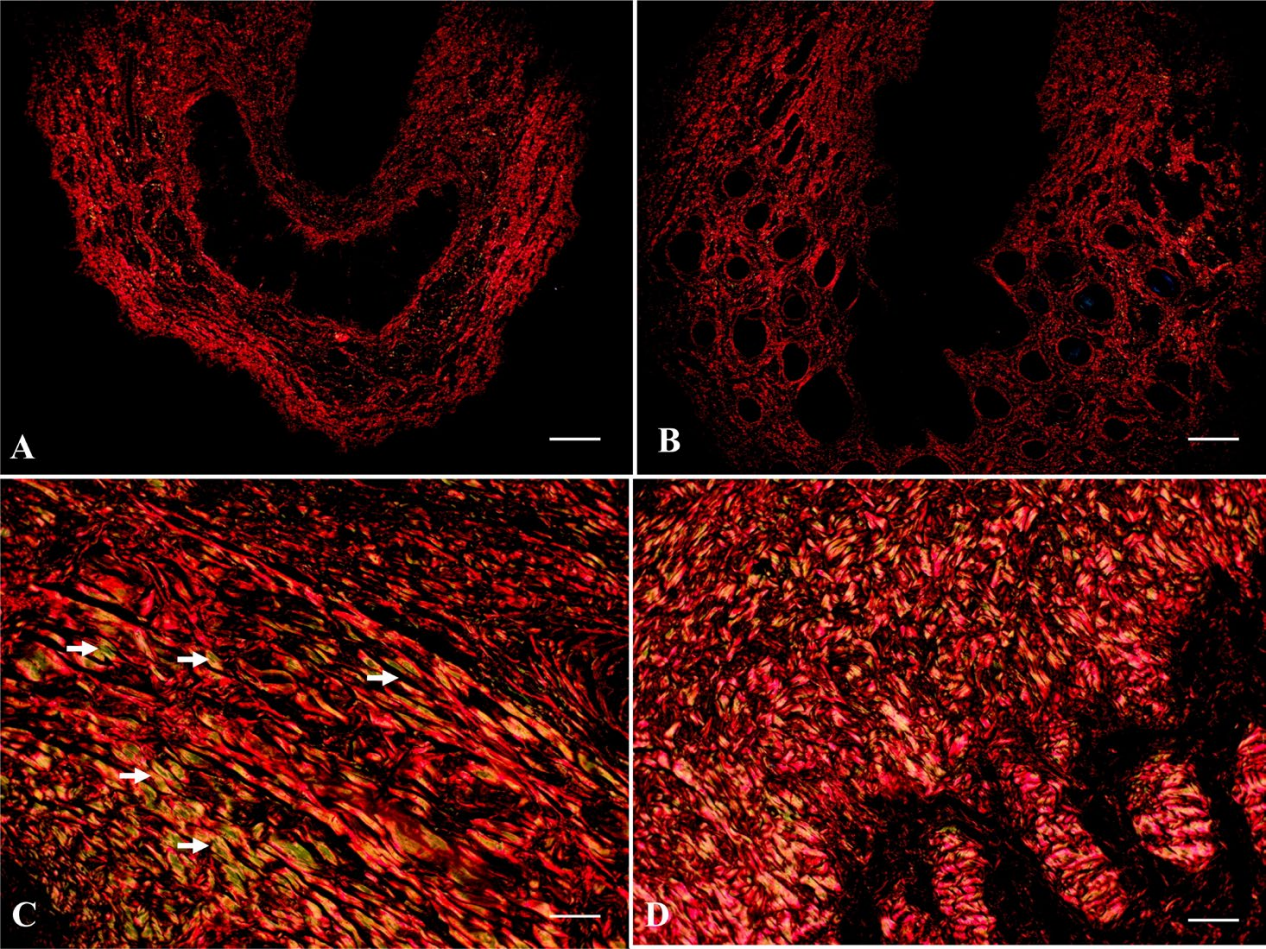
Histochemical characterisation of penile histology in giant anteaters using polarisation microscopy. (A) Collagen fibres in the penis of a young giant anteater. Picrosirius staining bar = 200 μm. (B) Collagen fibres in the corpus cavernosum of the penis of a young anteater. Picrosirius staining bar = 100 μm. (C, D) Collagen fibres type I and III (white arrow) in the penis of an adult. Picrosirius staining bar = 100 μm.

**FIGURE 4:**
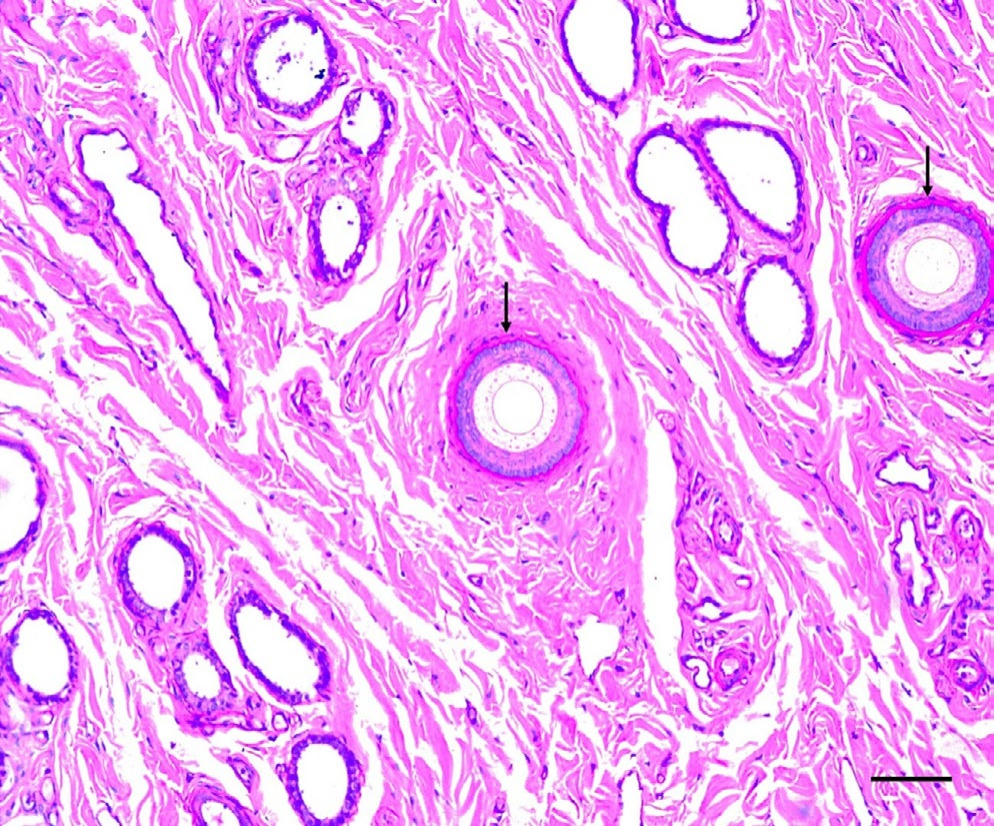
Histochemical characterisation using light microscopy revealed positive markings in the follicles of the penis of giant anteaters. PAS staining, bar = 50 μm.

Picrosirius staining shows the distribution of type I collagen fibres (red‐stained) throughout the organ stroma. Type I collagen fibres were less evident in the young erectile bodies (Figure 3A). Notably, the tunica albuginea of adults contained a high concentration of type I collagen (Figure 3C,D). Type III collagen fibres (stained green) were also observed, lining the corpora cavernosa and distributed throughout the connective tissue of the organ's stroma in adults (Figure 3C,D). A difference in collagen concentration was observed in the two adult participants; however, one participant was more senile than the other. Individual 3 (Table 1) showed a higher concentration of type III collagen fibres than type I, unlike individual 1 (Table 1), which was the youngest of the two animals in which type I collagen fibres were more prevalent than type III (Figure 4). PAS was used as another stain, and it was observed that the epithelial cells at the base of the hair follicles were rich in glycogen and positive for PAS (Figure 4).

**FIGURE 5:**
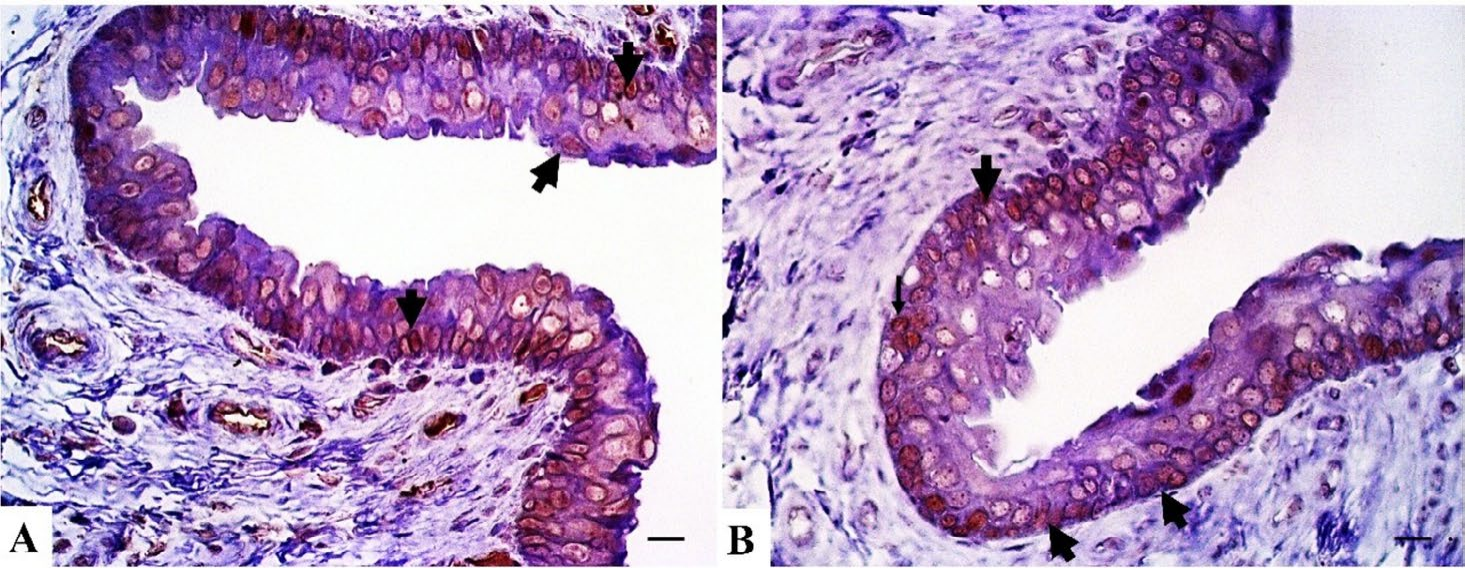
Immunohistochemical analysis of the giant anteater penis under light microscopy. (A) Androgen receptor nuclear immunolabelling (black arrow) in the urethral transitional epithelium. AR immunohistochemistry (IHC), bar = 20 μm. (B) Oestrogen receptor nuclear immunolabelling (black arrow) in the urethral transitional epithelium. ER IHC, bar = 20 μm.

Immunohistochemical analysis revealed intense positive nuclear staining in all cells of the transitional epithelium of the penile urethra and low or absent nuclear expression in the penile stromal cells in this species for the AR and ER (Figure 5).

## 4 Discussion

This study describes the morphological, histochemical and immunohistochemical characteristics of the giant anteater penis. Our results are compatible with what has already been described in the literature, for example, in terms of the absence of foreskin formation and the location of the two erectile bodies, the corpora cavernosa pair, corpus spongiosum and urethra in anteaters (Fromme et al. 2021; Rossi et al. 2012). These penile features differ from those of other mammals, including humans, in which the corpus cavernosum is located dorsally to the corpus spongiosum and urethra (Bartmann et al. 1991; Dyce et al. 2010; Junqueira and Carneiro 2013; Samuelson 2007). The giant anteater's vulva is commonly referred to as a ‘pseudopenis’. Some researchers have considered that giant anteaters lack sexual dimorphism, and this inability to distinguish between males and females by sight alone has led to the belief in parts of the population that only females exist, or that giant anteaters are hermaphrodites (Portante 1977; Lévi‐Strauss 1988; Bertassoni 2012). However, in our study, simple morphological inspection showed that the anatomy of the penis and vulva structures is different in the species. We believe that this was an important detail achieved within the study of the one vulva inspection in the present research.

One study observed a change in the organisation of collagen in the tunica albuginea of diabetic rabbits, in which there was a predominance of type III collagen fibres, unlike that described in the control group of rabbits without diabetes, in which type I collagen fibres were predominant (Abidu‐Figueiredo et al. 2010). Another study evaluated changes in the amount of collagen and elastic fibres according to age in the corpus spongiosum of the penis of rats and found that the extracellular matrix of the corpus spongiosum undergoes changes in its composition due to ageing, with both elastic and collagen fibres undergoing changes (Pigatto Filho et al. 2020). In this study, a decrease in the proportion of type I and III collagen fibres was observed, with an increase in type III collagen compared to type I collagen, increasing tissue fragility because type III fibres are not as resistant as type I fibres. Souza et al. (2012) evaluated the effects of chronic stress on the corpus cavernosum of the rat penis and found a decrease in smooth muscle fibres and an increase in connective tissue in animals subjected to chronic stress. In addition, a predominance of type I collagen fibres was observed in stressed animals compared to animals in the control group, in which there was a predominance of type III collagen fibres. In our study, we observed that collagen fibre distribution differed in the penis of young to adult giant anteaters, and we did not observe any microscopic alterations in the two cases with inflammatory infiltrates.

PAS staining can be used to diagnose benign and premalignant penile lesions, such as thickened basement membranes in the dermis (Sewell and Oxley 2019). In our study, we did not observe any dermal abnormalities; however, normal epithelial cells at the base of the hair follicles were positive for PAS staining.

Androgens and oestrogens are important in driving penile development; however, oestrogens can affect this development by interrupting androgen signalling, thus preventing penile growth and the normal closure of the urethra, leading to hypospadias during postnatal development (Govers et al. 2019). Another study evaluated the androgen‐responsive epithelium of the penis in mice, which was more persistent than that in humans. Some human penile cancers express hormonal receptors that are normally expressed in the fetus (Huang et al. 2020). In this study, we observed through immunohistochemistry that the giant anteater penis contains androgen and oestrogen receptors in adult and young subjects, as in mice.

We hope that this study will contribute to further research and that the information obtained will encourage the development of new tools and biotechnologies that can be applied to the reproduction of *Myrmecophaga tridactyla* , thereby contributing to the conservation of the species.

## 5 Conclusions

In conclusion, this study provides valuable information on the histological features of the penis of *Myrmecophaga tridactyla*.

## Author Contributions

**Fernanda Barthelson Carvalho de Moura** and **Victor Gustavo Santos Môra:** data curation; methodology; visualisation; validation; writing – review and editing; writing – original draft; investigation. **Natalia Camargo Faraldo**, **Maria Valeria de Toledo Rodovalho**, **Gabriel Correa de Camargo**, **Daniel Felipe Barrantes Murillo**, **Carlos Roberto Teixeira:** methodology; investigation; writing – review and editing. **Noeme Sousa Rocha** and **Tatiane Terumi Negrão Watanabe:** conceptualisation; methodology; writing – review and editing; funding acquisition; project administration; data curation. **Carlos Eduardo Fonseca‐Alves:** conceptualisation; data curation; methodology; visualisation; validation; writing – review and editing; writing – original draft; investigation; supervision.

## Conflicts of Interest

Tatiane Terumi Negrão Watanabe was employed at Antech Diagnostics and Mars Petcare Science and Diagnostics. The authors declare no potential conflicts of interest concerning the research, authorship or publication of this article.

## Data Availability

The data that support the findings of this study are available from the corresponding author upon reasonable request.
